# Lucid Dreaming, Nightmares, and Sleep Paralysis: Associations With Reality Testing Deficits and Paranormal Experience/Belief

**DOI:** 10.3389/fpsyg.2020.00471

**Published:** 2020-03-18

**Authors:** Kenneth G. Drinkwater, Andrew Denovan, Neil Dagnall

**Affiliations:** Department of Psychology, Faculty of Health, Psychology and Social Care, Manchester Metropolitan University, Manchester, United Kingdom

**Keywords:** lucid dreaming, dissociated experiences, REM, reality testing, paranormal experiences

## Abstract

Focusing on lucid dreaming, this paper examined relationships between dissociated experiences related to rapid eye movement (REM) sleep (lucid dreaming, nightmares, and sleep paralysis), reality testing, and paranormal experiences/beliefs. The study comprised a UK-based online sample of 455 respondents (110 males, 345 females, *Mean age* = 34.46 years, *SD* = 15.70), who had all previously experienced lucid dreaming. Respondents completed established self-report measures assessing control within lucid dreaming, experience and frequency of nightmares, incidence of sleep paralysis, proneness to reality testing deficits (Inventory of Personality Organization subscale, IPO-RT), subjective experience of receptive psi and life after death (paranormal experience), and paranormal belief. Analysis comprised tests of correlational and predictive relationships between sleep-related outcomes, IPO-RT scores, and paranormal measures. Significant positive correlations between sleep and paranormal measures were weak. Paranormal measures related differentially to sleep indices. Paranormal experience correlated with lucid dreaming, nightmares, and sleep paralysis, whereas paranormal belief related only to nightmares and sleep paralysis. IPO-RT correlated positively with all paranormal and sleep-related measures. Within the IPO-RT, the Auditory and Visual Hallucinations sub-factor demonstrated the strongest positive associations with sleep measures. Structural equation modeling indicated that Auditory and Visual Hallucinations significantly positively predicted dissociated experiences related to REM sleep, while paranormal experience did not. However, paranormal experience was a significant predictor when analysis controlled for Auditory and Visual Hallucinations. The moderate positive association between these variables explained this effect. Findings indicated that self-generated, productive cognitive-processes (as encompassed by Auditory and Visual Hallucinations) played a significant role in conscious control and awareness of lucid dreaming, and related dissociative sleep states (sleep paralysis and nightmares).

## Introduction

### Lucid Dreaming Background

Lucid dreaming is a dissociated state, which combines aspects of waking and dreaming (Schredl and Erlacher, 2004; Voss et al., 2009; LaBerge et al., 2018). Specifically, it denotes conscious awareness of dreaming during ongoing sleep (Baird et al., 2019). A central characteristic is that experiencers are typically able to signal their lucid state during dream periods using pre-agreed eye-movement signals (LaBerge, 1980; LaBerge et al., 2018). Concomitantly, lucid dreaming possesses consciousness-related features such as access to waking memories, increased insight and control, positive affect, body dissociation, and logical thought (LaBerge et al., 1981; Voss et al., 2009, 2018). Other criteria used to distinguish lucid dreams are memory of the waking state, sentience of freedom of decision, and full intellectual abilities (Tholey and Utecht, 1987; Lee, 2017). However, few lucid dreams include all of these features (Zink and Pietrowsky, 2013).

The concept of lucid dreaming pre-dates modern science as evinced by the work of ancient scholars (Baird et al., 2019). The modern conceptualization of lucid dreaming arose from Frederik van Eeden’s examination of his personal dream experiences. van Eeden (1913) defined lucid dreams as a state in which “…the reintegration of the psychic functions is so complete that the sleeper remembers day-life and his own condition, reaches a state of perfect awareness, and is able to direct his attention, and to attempt different acts of free volition” (pp. 149–150).

The development of physiological measurement and enhanced understanding of rapid eye movement (REM) sleep enabled researchers to produce empirical evidence that supported the existence of lucid dreaming and facilitated the development of objective measurement techniques. For instance, the ability to record pre-agreed eye movement sequences within lucid dreams became an established procedure (LaBerge et al., 1981, 2018).

Understanding of lucid dreaming has developed over recent years. Illustratively, Stumbrys et al. (2014) conducted a large-scale survey (*N* = 684) that identified important characteristics of lucid dreams. They found that lucid dreamers usually have their first experiences during adolescence, and these occur spontaneously. They noted also that the average lucid dream duration is about 14 min. In terms of phenomenology, lucid dreamers are typically active within their dreams and direct various actions (e.g., flying). Although, they are not always able to achieve their goals due to awakening, obstacles within the dream environment, or failing to recall intention (Stumbrys et al., 2014).

Incidence of lucid dreaming varies across studies as a function of methodology (researcher questions, classification criteria, type of data collection used, etc.) and sample type (see Saunders et al., 2016). A meta-analysis undertaken by Saunders et al. (2016) provides the best approximation of prevalence (number of individuals experiencing at least one lucid dream) and frequency (those reporting one or more lucid dreams per month). This estimated that 55% of adults have had at least one lucid dream in their lives, with 23% of adults experiencing lucid dreaming regularly (once per month or more).

### Individual Differences Related to Lucid Dreaming

Noting individual differences in prevalence and frequency, much research has focused on identifying the psychological variables that facilitate lucid dreaming. Notably, work examining the role of personality has found that the Big Five personality factors (openness to experience, conscientiousness, neuroticism, extraversion, and agreeableness) explain a small but substantial portion of variation (Hess et al., 2017). Specifically, Hess et al. (2017) found that openness to experience positively predicted lucid dreaming frequency, whereas agreeableness correlated negatively. Furthermore, controlling for nightmare frequency eliminated the relationship between neuroticism and lucid dreaming frequency. The openness findings concurred with Schredl and Erlacher (2004), who reported small significant relationships between lucid dreaming frequency, openness to experience, associated dimensions (thin boundaries, absorption, imagination), and openness facets of fantasy, aesthetics and feelings.

In addition to the Big Five personality factors, lucid dreaming correlates with specific personality characteristics (Blagrove and Tucker, 1994; Blagrove and Hartnell, 2000). For instance, frequent lucid dreamers (vs. non-lucid dreamers) score significantly higher on internal locus of control, need for cognition and creativity (Blagrove and Hartnell, 2000). Zink and Pietrowsky (2013) propose that these characteristics index cognitive complexity and flexibility. They also suggest a preference for self-focused attention, cognitive activity, and strong imaginative pursuits. Overall, these conclusions are consistent with studies that report self-reflectiveness and active control are integral features of lucid dreaming (Blagrove and Hartnell, 2000).

Noting this, Zink and Pietrowsky (2013) postulated that creativity plays a principal role in lucid dreaming. Indeed, Stumbrys and Daniels (2010) found that lucid dreaming contributed to problem solving in creative tasks. Alongside creativity, lucid dreaming correlates with related variables. Explicitly, fantasy proneness and absorption (Koffel and Watson, 2009). These constructs also relate to other sleep experiences (i.e., retrospective dream recall and dream salience; bizarreness, vividness, colorfulness, and impact of dreaming) (Koffel and Watson, 2009). Overall, related literature suggests that the correlated constructs of creativity, fantasy proneness and absorption represent a cognitive style based on intensive and absorptive imaginative involvement (Levin and Young, 2002).

### Reality Testing and Lucid Dreaming

Within the psychological literature, there exist different definitions of reality testing. The researchers used the conceptualization employed by the reality testing (IPO-RT) subscale of the Inventory of Personality Organization (IPO) (Lenzenweger et al., 2001). The IPO is a self-report measure that classifies personality pathology within clinical and non-clinical samples (Smits et al., 2009; Lenzenweger et al., 2012; Preti et al., 2015). The selection of the IPO-RT derived from the observation that the subscale indexes internally generated creative, imaginative and vivid mental sensations/imagery. Explicitly, the IPO-RT delineates reality testing as “the capacity to differentiate self from non-self, intrapsychic from external stimuli, and to maintain empathy with ordinary social criteria of reality” (Kernberg, 1996, p. 120). Accordingly, the IPO-RT focuses on information processing and provides an assessment of evaluative mechanisms (Langdon and Coltheart, 2000). Thus, high scores on the IPO-RT are indicative of a self-oriented, subjective information processing style, which indexes individual reliance on internally generated data, specifically intensive, absorptive imaginative involvement.

Noting the main features of lucid dreaming, and the fact that reality testing shares important attributes with lucid dreaming (creativity, inner focus, fantasy proneness, etc.) this paper examined the degree to which reality testing predicted lucid dreaming. Congruent with this perspective, researchers use the IPO-RT as an indirect, proxy measure of intuitive thinking style (Dagnall et al., 2017). This approach derives from the work of Epstein (1990, 1994), who developed cognitive-experiential self-theory, which differentiates experiential (fast, automatic, holistic, and characterized by proneness to generalization/association) and rational (slow, intentional, effortful, and logical) processing. In this context, high scores represent a preference for subjective, internally generated information and index greater tendency to reality testing deficits.

It is important to note that the IPO-RT samples a broad spectrum of cognitive-perceptual phenomena. Recognizing this, Irwin (2004) contended that the single factor solution depicted in the original paper represented an oversimplification of domain content. This applied to sleep research, implies that particular aspects of reality testing may be more predictive of lucid dreaming. Recognizing this, the present paper treated the IPO-RT as a multidimensional measure. The factorial structure selected derived from Dagnall et al. (2017), who identified four factors: hallucinations (auditory and visual), delusional thinking (beliefs contrary to reality), social deficits (difficulties reading social cues), and sensory/perceptual confusion (inability to understand feelings and sensations). These factors accounted for 55% of response variance and were conceptually congruent with the construct of reality testing within the IPO-RT (Bell et al., 1985; Caligor and Clarkin, 2010).

Subsequent psychometric evaluation of the IPO-RT by Dagnall et al. (2018) confirmed the presence of a bifactor structure consisting of a general dimension encompassing the four distinct, but inter-correlated sub-factors. Consideration of the role that sub-factors of reality testing play in lucid dreaming provides a more precise, fine-grained understanding of the cognitive-perceptual conditions involved in lucid dreams.

### Lucid Dreaming and Paranormal Experiences/Beliefs

In addition to the IPO-RT, the present study included paranormal measures (i.e., belief and experience). Previous work informed this decision. Firstly, Glicksohn (1990) found that belief in the paranormal correlated positively with subjective paranormal experiences, which in turn were associated with incidence of at least one altered state of consciousness and level of absorption. Based on this finding, Glicksohn (1990) concluded that altered states of consciousness often reflect psychological elements of the relationship between paranormal belief and experience. Pertinent to the present paper, altered states of consciousness indexed phenomena related to the sleep–wakefulness continuum: lucid dreams, transitions between sleep and wakefulness (hypnagogic and hypnopompic states), and out-of-the-body experience (i.e., the experience of separation from the physical body). Moreover, paranormal experience correlated with incidence of lucid dreaming.

Secondly, although the direct relationship between paranormal belief and lucid dreaming is weak (see Glicksohn, 1990; Denis and Poerio, 2017), studies generally observe significant positive relationships between paranormal belief and major constructs associated with lucid dreaming. Notably, openness to experience (Smith et al., 2009), creativity (Irwin, 1993; Thalbourne and Delin, 1994; Thalbourne, 2005), and boundary thinness as measured by transliminality (Dagnall et al., 2010c). Transliminality denotes hypersensitivity to psychological material (Thalbourne and Maltby, 2008). Particularly, it is “a hypothesized tendency for psychological material to cross (trans) thresholds (limines) into or out of consciousness” (Thalbourne and Houran, 2000, p. 853).

Finally, belief in the paranormal correlates positively (moderately) with proneness to reality testing deficits (Drinkwater et al., 2012; Dagnall et al., 2014). Cumulatively, these findings suggest relationships between lucid dreaming, reality testing deficits and experience of the paranormal.

### Other Dissociated Experiences Related to Rapid Eye Movement Sleep (Sleep Paralysis and Dreaming) and Paranormal Experiences/Beliefs

With relevance to the present study, it is worth noting that Glicksohn (1990) found that only paranormal experience predicted lucid dreaming. Cognizant of this, the authors focused on commonly encountered ‘productive’ psychic experiences (see Glicksohn, 1990; Dagnall N.A. et al., 2016). Specifically, receptive forms of psi (telepathy, precognition, premonition, and remote viewing) and communication with spirits (contacting the deceased, psychic ability, mediumship, and spiritualism). Thematically, these phenomena comprise the mental transmission and reception of information via unknown powers or forces, and are concomitant with an open and intuitive approach to experiences (Schmeidler, 1985).

Alongside lucid dreaming, the authors included other dissociated experiences related to REM sleep (i.e., sleep paralysis and dreaming). Sleep paralysis was justified because it correlates positively with lucid dreaming (Denis and Poerio, 2017), and experiencers frequently report concomitant unusual/anomalous perceptions and sensations (Denis et al., 2018). Sleep paralysis combines elements of wakefulness and REM sleep, characterized by the inability to perform voluntary movements during sleep onset or awakening (i.e., the sleeper is “immobilized” yet perceptually awake) (see American Academy of Sleep Medicine, 2014; Jalal, 2018).

A key feature of sleep paralysis relevant to the current paper was accompanying hallucinations (strong visual imagery) (Spanos et al., 1995). These often take the form of uncanny “ghost-like” experiences and evoke extreme fear reactions (Jalal, 2018). Cheyne places these into three categories: intruder (sense of evil presence and multi-sensory hallucinations of intruder), incubus (feeling of pressure on the chest, suffocation, and physical pain), and vestibular-motor (feature illusory-movement and out-of-body experiences) (Cheyne et al., 1999b; Cheyne, 2003). Intruder and incubus hallucinations typically co-occur and are accompanied by fear, whereas vestibular-motor hallucinations are more positive (Cheyne, 2003).

As with lucid dreaming, studies report that personality factors influence occurrence of sleep paralysis. Particularly, thinner personality boundaries correlate with pleasant sleep paralysis, and individuals with higher absorption demonstrate greater propensity to sleep paralysis with hallucinations (Lišková et al., 2016).

Moreover, Denis and Poerio (2017) found that sleep paralysis and lucid dreaming were associated with belief in the paranormal. Denis and Poerio (2017) suggest openness to experience explains this connection. In addition, imaginal capacity plays an important role in both lucid dreaming and sleep paralysis. Relatedly, the strongest predictor of sleep paralysis episodes was nightmares (Spanos et al., 1995; Lišková et al., 2016). Nightmares are extremely frightening dreams from which the person is directly awakened (Spoormaker et al., 2006). Although, the relationship between nightmares and lucid dreaming is complex and difficult to establish, nightmare prevalence and distress is also associated with higher levels of fantasy proneness, and psychological absorption. Noting this, the present study considered nightmares together with lucid dreaming and sleep paralysis for completeness.

### The Present Study

The linkage between other dissociated experiences related to REM sleep and paranormal experiences/beliefs suggests that factors share common features, which merits further investigation. Certainly, previous studies such as Denis and Poerio (2017) have reported weak associations between paranormal belief, dissociative experiences, lucid dreaming, sleep paralysis, daydreaming, and imagery. Hence, the present study extended understanding of the relationship between cognitive-perceptual personality factors by examining the extent to which reality testing and paranormal belief/experience predicted lucid dreaming and sleep-related phenomena (i.e., sleep paralysis and dreaming). The inclusion of reality testing derived from the constructs focus on intra-psychic activity and overlap with factors linked to lucid dreaming (i.e., creativity, imagination, fantasy proneness, and absorption). These elements link with consciousness and belief/experience of the paranormal.

Accordingly, the authors hypothesized that reality testing would correlate with belief in and experience of the paranormal, and predict lucid dreaming, sleep paralysis and nightmares. Given that the present study included only respondents who experienced lucid dreaming and focused on control of lucid dreaming, the authors tentatively anticipated correlations between sleep-related factors. This postulation resulted from the view that experiencers of lucid dreaming possess a greater awareness of sleep-related phenomena, especially when experiences reference perception of visual imagery and imagined sensations.

Consistent with previous work and the supposition that ‘experience’ more directly indexes acceptance of the existence of paranormal forces than belief, the researchers posited that only paranormal experience (not belief) would predict lucid dreaming. This notion is congruent with attributional models, which regard the labeling of anomalous experiences as ‘paranormal’ as the final process stage (see Irwin et al., 2013). Finally, the inclusion of a range of sleep-related measures enabled the researchers to determine whether reality testing and paranormal measures were similarly predictive of lucid dreaming, sleep paralysis, and nightmares.

## Materials and Methods

### Procedure

Prior to participation, potential respondents received background information. This stated the nature of the study and outlined ethics. Only respondents providing informed consent received the materials booklet. Instructions asked respondents to carefully read, answer all questions, and take their time. Participants worked through the measures at their own pace and there was no maximum time limit. To prevent order effects questionnaire position rotated.

Within the present study, data collection occurred at one point in time. Such cross-sectional designs are frequently criticized because they can result in common method variance (Spector, 2019). To prevent common method variance, the researchers employed procedural remedies (Krishnaveni and Deepa, 2013). Firstly, the study brief and scale instructions emphasized that each measure assessed a different construct. This created psychological distance between the scales. Separation strategies, such as this have previously successfully reduced common method variance (Podsakoff et al., 2003). Secondly, the study brief provided information intended to reduce the potential for social desirability effects and evaluation apprehension by stating that there were no correct answers, and advising respondents that they should answer questions honestly.

### Participants

The study sample comprised 455 respondents, (Mean age, *M*) = 34.46 years, *SD* = 15.70, range 18–77. There were 110 males (24%), *M* = 25.31 years, *SD* = 9.56, range 19–77; and 345 females (76%), *M* = 28.00 years, *SD* = 11.76, range 18–75. For all variables, skewness and kurtosis values were within the recommended range of −2.0 to +2.0 (Byrne, 2010). Participant recruitment was via emails to university staff/students and local stakeholders (businesses, leisure and vocational/sports classes). If potential participants had not experienced lucid dreams or were younger than 18 years of age participation discontinued. Other studies have also focused on respondents who have experienced sleep-related phenomena (see Lišková et al., 2016; study of sleep paralysis). These factors were the only exclusion criteria.

### Measures

#### Dissociated Experiences Related to Rapid Eye Movement Sleep

##### Lucid dreaming

Four items indexed subjective reporting of lucid dreaming (frequency and control). In order to confirm that respondents understood what lucid dreams were the first item acted as a screening check. This included a brief definition preceded by a rating scale, “During lucid dreaming, one is—while dreaming—aware of the fact that one is dreaming. It is possible to deliberately wake up or to control the dream action or to observe passively the course of the dream with this awareness” (Snyder and Gackenbach, 1988).

Frequency was assessed using an eight-point rating scale (0 = never, 1 = less than once a year, 2 = about once a year, 3 = about two to four times a year, 4 = about once a month, 5 = about two to three times a month, 6 = about once a week, 7 = several times a week) (Schredl and Erlacher, 2004; Stumbrys and Erlacher, 2017). This item ensured that respondents had experienced lucid dreams. Respondents reporting lucid dreams rated the extent (in percentages) they were able to maintain conscious awareness for a sufficiently long period of time; completely control their dream body (movements and actions); and design their dream surroundings (to make landscape or environment and occurring dream characters to appear, disappear, or change) (Stumbrys and Erlacher, 2017). In this study, internal consistency was good for this scale, α = 0.81.

##### Nightmares

Two items assessed the degree to which respondents experienced and recalled nightmares (Schredl et al., 2016). A brief definition of nightmares appeared prior to scale completion, “A nightmare is a vivid dream that is frightening and disturbing, the events of which you can remember clearly and in detail when you wake up.”

The first item asked, “How often do you experience nightmares?” Respondents answered using an eight-point Likert scale (0 = never, 1 = less than once a year, 2 = about once a year, 3 = about two to four times a year, 4 = about once a month, 5 = about two to three times a month, 6 = about once a week, 7 = several times a week). The second item asked, “How distressing are your nightmares?” was measured using a five-point scale (0 = not at all distressing, 1 = not that distressing, 2 = somewhat distressing, 3 = quite distressing, and 4 = very distressing). The third item assessed recall, “How often do you wake up and recall a dream” (Schredl, 2004). Participants responded via a seven-point Likert scale (1 = never, 2 = less than once a month, 3 = about once a month, 4 = twice or three times a month, 5 = about once a week, 6 = several times a week, 7 = almost every morning). Alpha reliability for this measure was satisfactory, α = 0.68.

##### Movement

A final single item measured respondent experience of sleep paralysis, “Sometimes when falling asleep or when waking from sleep, people may experience a brief period of inability to move, even though they are fully conscious and awake. How often do you recall this experience?” (Cheyne et al., 1999a). Participants responded via a four-point Likert scale (1 = never, 2 = once, 3 = two to five times, and 4 = more than five times).

#### The Reality Testing Subscale of the Inventory of Personality Organization (IPO-RT)

The IPO-RT (Lenzenweger et al., 2001) assesses the ability to differentiate self from non-self, intrapsychic from external stimuli, and to maintain empathy with ordinary social criteria of reality (Kernberg, 1996). This perspective derives from an information-processing approach to belief generation (see Langdon and Coltheart, 2000). Consequently, researchers use the IPO-RT to assess proneness to reality testing deficits (Irwin, 2004; Dagnall et al., 2017). Particularly, as an index of the tendency to engage in subjective-intuitive thinking (Denovan et al., 2017). The IPO-RT comprises 20-items that appear as statements (e.g., “I believe that things will happen simply by thinking about them”). Respondents specify their level of agreement on a five-point Likert scale. Possible responses range from 1 = never true to 5 = always true. Summation of item totals produces scores between 20 and 100. Higher scores indicate propensity to reality testing deficits. Previous research has established that the IPO-RT is psychometrically robust. Explicitly, good internal consistency, test–retest reliability, and construct validity (Lenzenweger et al., 2001). In this study, good internal consistency existed, α = 0.92.

#### Paranormal Measures

##### Manchester Metropolitan University New (MMU-N)

This study used the MMU-N (Dagnall et al., 2010a, b) to assess belief in the paranormal in preference to the Revised Paranormal Belief (Tobacyk and Milford, 1983) and Australian Sheep-Goat (Thalbourne and Delin, 1993) scales because the MMU-N measures a broader range of beliefs, and samples these in greater depth. The MMU-N provides both overall and dimensional, sub factor scores (i.e., hauntings, superstitions, religious belief, alien visitation, extrasensory perception, psychokinesis, astrology, and witchcraft) (Dagnall et al., 2010a, b). These subscales are conceptually coherent, possess good face validity and are composed of items clearly related to the assigned factor label. The MMU-N comprises 50-items presented as statements (e.g., ‘there is a devil’ and ‘poltergeists exist’) to which participants respond using a seven-point Likert scale (ranging from 1, strongly disagree, to 7, strongly agree). Both subscales and the overall measure possess good to excellent external reliability (Dagnall et al., 2010a). The measure has featured in published studies, where it has demonstrated good concurrent validity. In the current study, this scale evidenced good reliability, α = 0.96.

##### Paranormal Experience

A series of items asked respondents whether they had genuinely experienced paranormal/psychic phenomena (i.e., communication with the dead, psychic, mediumship, spiritualism, telepathy, precognition, premonition, and remote viewing). These items represented core subjective experiences related to receptive psi and life after death (see Drinkwater et al., 2013, 2017a). To ensure that respondents understood what each phenomenon was, a definition appeared within each category. For example, ‘Mediums receive and relay information from deceased people to the living. In the context of this definition, have you ever personally experienced mediumship? ‘Summation of category scores produced an overall experience total. Scores ranged from 0 to 8, with higher scores indicating greater experience of paranormal/psychic phenomena. This method of measuring experienced paranormal/psychic phenomena is well-established (Dagnall et al., 2019). Satisfactory alpha reliability existed for this measure, α = 0.74.

For analysis, the researchers used mean total score for each variable (see Table 1).

**TABLE 1:** Means, standard deviations and correlations for all study variables (*N* = 453).

**Variable**	***M***	***SD***	**Skew**	**Kurt.**	**1**	**2**	**3**	**4**	**5**	**6**	**7**	**8**	**9**	**10**
1. IPO-RT	40.93	13.37	0.85	0.64		0.89**	0.76**	0.66**	0.92**	0.49**	0.37**	0.19**	0.18**	0.18**
2. AVH	11.96	4.81	0.88	0.28			0.56**	0.52**	0.74**	0.47**	0.41**	0.20**	0.17**	0.18**
3. SD	7.02	2.94	0.97	0.62				0.33**	0.65**	0.22**	0.19**	0.09	0.04	0.16**
4. Confusion	8.51	2.61	0.18	–0.29					0.48**	0.31**	0.09*	0.12*	0.25**	0.07
5. DT	13.42	5.56	0.92	0.55						0.50**	0.38**	0.17**	0.15**	0.17**
6. PB	170.46	53.32	–0.83	–0.50							0.52**	0.04	0.18**	0.09*
7. PExp	1.83	1.87	1.13	0.76								0.11*	0.12*	0.18**
8. LD	56.26	71.44	1.39	1.15									0.25**	0.23**
9. Nightmare	11.81	3.48	–0.09	–0.26										0.24**
10. SP	1.92	1.08	0.70	–0.98										

IPO-RT, Inventory of Personality Organization-Reality Testing subscale; AVH, Auditory and Visual Hallucinations; SD, social deficits; DT, delusional thinking; PB, paranormal belief; PExp, paranormal experience; LD, lucid dreaming; SP, sleep paralysis. *p < 0.05, **p < 0.01 (also less than the Benjamini and Hochberg adjusted critical *p*-values).

### Ethics Statement

As preparation for a grant bid (October 2018), the researchers gained ethical endorsement for a series of studies examining psychological and neuropsychological factors associated with self-professed psychic ability/mediumship. Following formal submission, the Director of the Research Institute for Health and Social Care and the Manchester Metropolitan University Faculty of Health, Psychology and Social Care Ethics Committee granted ethical approval.

### Data Analysis

Data screening occurred prior to computation of descriptive statistics (means, *SD*s, and correlations) and model testing. Model testing via AMOS26 (IBM SPSS) comprised structural equation modeling, which is a sophisticated analytic technique that tests hypotheses by computing the weight of standardized regression paths between variables of interest (depicted as latent variables). Structural equation modeling incorporates measurement error in its model estimation, and utilizes fit indices to evaluate the extent to which observed data corresponds with proposed, conceptual models.

Preceding model testing, confirmatory factor analysis examined the adequacy of each study instrument and a measurement model scrutinized interactions between latent variables and accompanying outcomes. A structural model subsequently assessed hypothesis-driven relationships among latent variables (Anderson and Gerbing, 1988). Specifically, the degree to which reality testing and paranormal belief and/or experience predicted sleep-related outcomes (lucid dreaming, sleep paralysis, and nightmares).

A range of indices determined model fit, specifically absolute fit indices (chi-square statistic, root-mean-square error of approximation, RMSEA; standardized root-mean-square residual, SRMR), and relative fit indices (comparative fit index, CFI; incremental fit index, IFI). Chi-square considers the extent to which a model reproduces data, with non-significant *p*-values indicative of good fit. However, chi-square frequently rejects models informed by large samples due to its sensitivity to sample size. Consequently, other indices require inspection (Kline, 2010).

Root-mean-square error of approximation assesses the distance between the reproduced covariance matrix and the sample-based covariance matrix, and includes a 90% confidence interval (CI) to judge precision of fit. SRMR indexes the average of standardized residuals between hypothesized and actual covariance matrices (Cangur and Ercan, 2015). RMSEA and SRMR statistics of 0.08–1.0, 0.06–0.08 and ≤0.05 indicate marginal, satisfactory and good fit (Browne and Cudeck, 1993). Relative fit indices compare the performance of a tested model to a null model (also called an ‘independence’ model) (Ching et al., 2014). Values above 0.90 represent good fit (Hu and Bentler, 1999).

## Results

### Descriptive Statistics

Descriptive statistics and correlations appear in Table 1 alongside univariate kurtosis and skewness data. All values fell within the recommended range of −2 to +2 (Byrne, 2010). Given a large number of correlations existed, for comparison purposes adjustment to the significance level occurred using a sequential method suggested by Benjamini and Hochberg (1995); demonstrated by Williams et al. (1999). In this, ranking of *p*-values (from smallest to largest) takes place, resulting in adjusted critical *p*-values for statistical inference, according to the formula of I/K × 0.05 (i.e., observed *p*-value rank/number of comparisons × level of significance). All comparisons utilized the 0.05 significance level. This method regulates the false positive rate, ensuring that no more than 5% of results identified as significant are in the wrong direction.

Using the Benjamini and Hochberg procedure, total IPO-RT (reality testing) evidenced small to medium (albeit significant) correlations with paranormal belief, experience, lucid dreaming, nightmares, and sleep paralysis. The Auditory and Visual Hallucinations subfactor of reality testing (compared with other subfactors) demonstrated the strongest associations with paranormal belief, experience, lucid dreaming, nightmares, and sleep paralysis. Of the paranormal measures, paranormal experience correlated most strongly with these outcomes. This result was consistent with study expectations and previous literature (Glicksohn, 1990). Accordingly, subsequent analyses focused on Auditory and Visual Hallucinations and paranormal experience. Given small correlations existed between Auditory and Visual Hallucinations and paranormal experience with lucid dreaming and nightmares, these variables had relatively low predictive value in the structural model.

### Confirmatory Factor Analyses

Confirmatory factor analysis occurred for each selected scale. Research indicates that Auditory and Visual Hallucinations is an intercorrelated but distinct unidimensional subfactor of IPO-RT (Dagnall et al., 2018). Paranormal experience also comprised one factor because the variable derived from experiences that were similar in theme and response scale. These indexed the most commonly reported attributes of paranormal experience (receptive psi and life after death) (see Drinkwater et al., 2013, 2017b). A two-factor correlated model examined lucid dreaming and nightmares (dreaming) given these constructs share semantic similarities (i.e., relate to types of dreaming). Confirmatory factor analysis excluded item 1 of lucid dreaming because the purpose of this item was to screen participants for inclusion in the study.

Prior to confirmatory factor analysis, data screening using Mardia’s test (Mardia, 1970) for selected study measures (Auditory and Visual Hallucinations, paranormal experience, lucid dreaming, and nightmares) indicated multivariate non-normality. Specifically, for Auditory and Visual Hallucinations multivariate kurtosis equaled 24.29 (critical ratio = 26.38); paranormal experience multivariate kurtosis = 41.25 (critical ratio = 34.71); and Dreaming (i.e., lucid dreaming and nightmares) multivariate kurtosis = 19.14 (critical ratio = 20.79). Consequently, subsequent confirmatory factor analyses utilized bootstrapping (1,000 resamples) to generate accurate bias-corrected model estimates (at the 95% confidence level). Nevitt and Hancock (2001) established that naïve bootstrapping is a sound alternative to other maximum likelihood robust approaches (e.g., Satorra–Bentler chi-square), and functions well even in instances of significant non-normality.

The Auditory and Visual Hallucinations unidimensional model indicated good fit on all indices but RMSEA, which reported marginal fit, χ^2^ (8, *N* = 453) = 44.02, CFI = 0.97, IFI = 0.97, SRMR = 0.03, RMSEA = 0.10 (CI of 0.07 to 0.13). All items loaded greater than 0.32. The unidimensional solution for paranormal experience evidenced good fit on CFI, IFI, and SRMR, and satisfactory RMSEA, χ^2^ (19, *N* = 453) = 60.52, CFI = 0.94, IFI = 0.94, SRMR = 0.05, RMSEA = 0.07 (CI of 0.05 to 0.09). All items demonstrated factor loadings greater than 0.32, but item 8 (0.24). The correlated two-factor model for Dreaming reported good fit overall, χ^2^ (8, *N* = 453) = 7.87, CFI = 1.0, IFI = 1.0, SRMR = 0.02, RMSEA = 0.01 (CI of 0.00 to 0.05). High factor loadings (above 0.5) existed for all items.

### Structural Equation Modeling

Consistent with the study hypotheses, the structural model tested the notion that Auditory and Visual Hallucinations and paranormal experience correlated positively and were predictive of greater levels of lucid dreaming, nightmares, and sleep paralysis. Prior to model testing, data screening (i.e., Mardia’s test; Mardia, 1970) indicated multivariate non-normality, as multivariate kurtosis = 120.03 (critical ratio = 41.09). Similar to confirmatory factor analyses, structural equation modeling utilized bootstrapping with 1,000 resamples.

**FIGURE 1:**
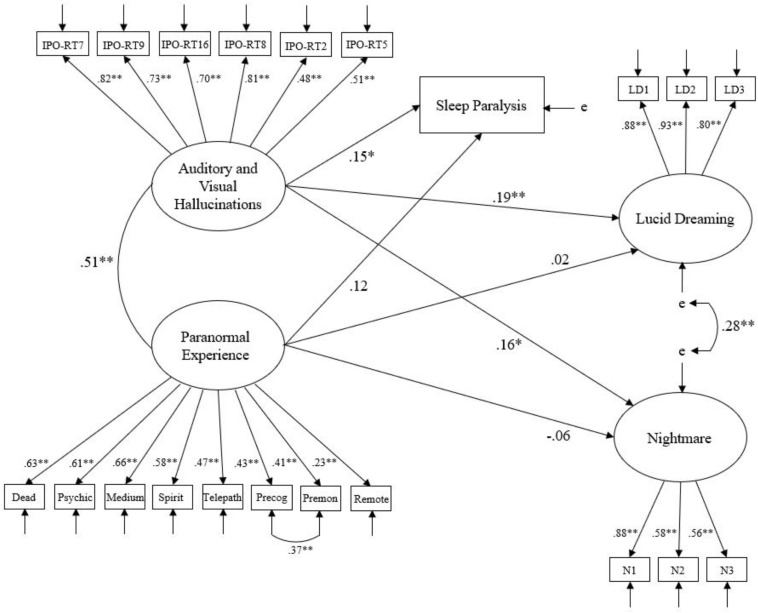
Model 2 – Hypothesized structural relationships between Auditory and Visual Hallucinations, paranormal experience, lucid dreaming, nightmares, and sleep paralysis. Ellipses indicate latent variables, squares indicate measured variables, and ‘e’ represents error of measurement. Lines between latent variables represent standardized coefficients; ^∗^*p* < 0.05, ^∗∗^*p* < 0.01.

A test of the measurement model (which depicted latent variables as correlated) suggested good relative fit and satisfactory absolute fit, χ^2^ (180, *N* = 453) = 476.56, CFI = 0.91, IFI = 0.91, SRMR = 0.06, RMSEA = 0.06 (CI of 0.05 to 0.07). A test of the hypothesized model (Model 1) depicting predictive relations from Auditory and Visual Hallucinations and paranormal experience to lucid dreaming, nightmares, and sleep paralysis indicated good relative and satisfactory absolute fit, χ^2^ (181, *N* = 453) = 451.27, CFI = 0.92, IFI = 0.92, SRMR = 0.06, RMSEA = 0.06 (CI of 0.05 to 0.06). Computing a new model (Model 2; Figure 1) and correlating error terms between paranormal experience items 6 and 7 (‘Precognition’ and ‘Premonition’) resulted in good fit on all indices but SRMR, which reported satisfactory fit, χ^2^ (180, *N* = 453) = 392.59, CFI = 0.93, IFI = 0.93, SRMR = 0.06, RMSEA = 0.05 (CI of 0.04 to 0.06). Statisticians caution against correlating error terms, unless appropriate justification exists (Byrne, 2010). In this case, both items belonged to the same scale and indexed a ‘sensation’ concerning perception of future events. Comparing the Akaike Information Criterion (AIC) of Model 1 (593.27) and Model 2 (536.59) revealed that Model 2 offered a superior fit to the data, given a lower value existed.

Inspection of standardized regression paths revealed that Auditory and Visual Hallucinations positively predicted lucid dreaming (β = 0.19, *p* = 0.003), nightmares (β = 0.16, *p* = 0.024), and sleep paralysis (β = 0.15, *p* = 0.016). Auditory and Visual Hallucinations also demonstrated a moderate positive correlation with paranormal experience (0.51, *p* < 0.001). However, paranormal experience did not significantly predict either outcome (lucid dreaming β = 0.02, *p* = 0.785; nightmares β = -0.06, *p* = 0.449; sleep paralysis β = 0.12, *p* = 0.098). An alternative model (Model 3) constrained the regression paths from Auditory and Visual Hallucinations to lucid dreaming outcomes to zero, thereby examining the influence of paranormal experience whilst controlling for Auditory and Visual Hallucinations. Although this model reported weaker fit compared with Model 2 (i.e., a higher AIC of 600.27), paranormal experience significantly predicted lucid dreaming (β = 0.16, *p* = 0.018) and sleep paralysis (β = 0.22, *p* = 0.002), but not nightmares (β = 0.09, *p* = 0.160). These findings inferred that Auditory and Visual Hallucinations was a significant positive predictor of lucid dreaming and its related facets, whereas paranormal experience was not. However, paranormal experience was a significant predictor when marginalizing the influence of Auditory and Visual Hallucinations. In addition, paranormal experience, and Auditory and Visual Hallucinations demonstrated a positive association with one another.

## Discussion

Examination of zero-order correlations revealed weak positive relationships between proneness to reality testing deficits (IPO-RT) and sleep-related variables (lucid dreaming, nightmares, and sleep paralysis). Explicitly, higher levels of self-oriented, subjective information processing style were associated with greater perceived control within lucid dreams, Nightmare experience and recall, and incidence of sleep paralysis. Although as predicted, paranormal measures positively correlated with proneness to reality testing deficits, relationships between belief and experience and sleep measures varied as a function of dissociated state. Specifically, paranormal belief correlated weakly with sleep paralysis and nightmares. Whereas, paranormal experience demonstrated similar weak relationships with lucid dreaming and sleep paralysis.

These outcomes aligned largely with previous research. Notably, Glicksohn (1990) who reported positive relationships between paranormal belief and subjective paranormal experience, and between subjective paranormal experience and lucid dreaming. Furthermore, Glicksohn (1990) also observed that paranormal belief was not associated with lucid dreaming. Differential relationships between lucid dreaming and paranormal factors within the current paper support the notion that ‘experience’ is a better predictor of lucid dreaming (conscious awareness and control) than belief. In the context of this article, this makes intuitive and conceptual sense because experiences focused on perception of productive phenomena (i.e., receptive psi and life after death; paranormal experience).

Paranormal explanations notwithstanding, from a psychological perspective experience(s) directly inform conclusions about the existence of supernatural forces (Irwin et al., 2013), and indirectly tap into creative, imaginative and control elements of consciousness. Contrastingly, beliefs do not require an experiential basis. Accordingly, they are abstract and less tangible than subjective paranormal experiences.

This supposition is consistent with previous work that found that reporting of spontaneous paranormal experiences was associated with openness to and exploration of psychological space (Holt et al., 2004; Drinkwater et al., 2017a). This is also congruent with the finding that internal sensitivity predicts propensity to psi experiences (Honorton, 1972). In turn, these factors may also explain in part the relationship between paranormal experience and lucid dreaming.

Examination of the predictive model provided further insights into the relationships between lucid dreaming, reality testing and paranormal experience. Although, paranormal experience correlated moderately with Auditory and Visual Hallucinations, it did not significantly predict nightmares and sleep paralysis. Controlling for Auditory and Visual Hallucinations resulted in significant predictive relationships between lucid dreaming, nightmares, and sleep paralysis. Given that Auditory and Visual Hallucinations demonstrated positive significant relationships with lucid dreaming, nightmares, and sleep paralysis, it is likely that this explained the majority of the variance when predicting the sleep-related outcomes.

With regard to dissociated experiences related to REM sleep, the emergence of Auditory and Visual Hallucinations as the major factor IPO-RT facet makes conceptual sense. Auditory and Visual Hallucinations possesses thematic correspondence with lucid dreaming (i.e., fantasy proneness and creativity) and links to constructs related to sleep paralysis and nightmares (i.e., hallucinations and strong visual imagery; Spanos et al., 1995). Hence, examining IPO-RT subfactors in the current study provided theoretical insights, which further understanding of the connection between lucid dreaming control and cognitive-perceptual individual differences arising from thinking style. Specifically, that the productive, ‘creative’ elements of reality testing linked to fantasy proneness explain the construct’s association with lucid dreaming. Other elements of reality testing (i.e., social deficits, confusion, and delusional thinking) make no significant contribution to lucid dreaming control. The finding that paranormal experience predicted lucid dreaming in the absence of Auditory and Visual Hallucinations accords with Glicksohn (1990).

Considering the content of sleep-related measures, lucid dreaming items were highly associated, whereas nightmare items demonstrated only weak and moderate relationships. This pattern of results indicated that aspects of lucid dreaming (maintaining conscious awareness, dream body control and design of dream surroundings) were more coherent and closely aligned than features of nightmares (frequency, distress, and dream recall). This was compatible with item level content, which in the case of nightmares sampled a spectrum of construct content. Sleep paralysis because it indexed frequency, rather than intensity and/or content, correlated weakly across lucid dreaming and nightmare items.

### Limitations and Suggestions for Future Research

A potential limitation of the present study was the use of self-report measures to assess dissociated experiences related to REM sleep. Although this is a well-established and frequently used approach, critics have questioned the accuracy of measurement instruments, particularly the degree to which they provide valid insights into complex cognitive-perceptual processes. In the context of sleep, there is evidence that suggests that self-report measures provide valid snapshots of sleep-related behaviors.

For instance, Biddle et al. (2015) found that self-reports for habitual sleep duration and onset time were effective compared to an objective measure (i.e., at least 7 days of actigraphy monitoring) within large-scale studies. However, they also found that indifferences, such as those observed in clinically heterogeneous samples could produce biased estimates. In such circumstances, the use of objective measures is necessary. Within the present study, there was no evidence of systematic bias in sleep behavior. Hence, it is reasonable to assume that the self-report measures provided reasonably valid insights into factors related to incidence and frequency.

Moreover, there remains concerns about the extent to which self-report measures provide accurate assessments of reality testing (Denovan et al., 2017). Reality testing is a complex cognitive-perceptual factor that involves both knowledge of and control of cognition (Larkin, 2009; Schneider and Artelt, 2010). These underlying mechanisms are not easy to assess consciously. From this perspective, the IPO-RT indexes subjective awareness of reality testing errors. The reflective, spontaneous evaluation of reality testing decisions means that judgments may often lack veracity and/or comprehension.

This a problem that applies to cognitive functions generally. Accordingly, researchers often report weak relationships between subjective and objective measures of cognitive performance (Reid and MacLullich, 2006; Buelow et al., 2014). Noting this, future studies may wish to assess reality testing via concurrent measures to ensure that the outcomes reported in this article do not reflect an artifact of the measure used. Although, it is worth noting that the IPO-RT has proved psychometrically robust and is commonly employed by researchers. Generally, the use of self-report measures facilitate studies such as the present one because they are expedient, easy to administrate, accessible, possess wide reach, easy to score, and do not draw upon researcher assessments (Bell et al., 1985).

Despite the robust methodology of the present study and its outcomes being consistent with corresponding research, there are potential limitations that restrict extrapolation of findings. One foremost concern centres on the use of a cross-sectional design, where data collection occurred at one point in time. Critics point out that it is impossible to establish causality via cross-sectional designs. This prevents definitive conclusions because outcomes may result from other unaccounted variables.

In addition to this, observed relationships were small and require cautious interpretation. This issue is not unique to the present study, but is a problem inherent within studies examining relationships between sleep-related factors and personality generally (see Denis and Poerio, 2017; Aviram and Soffer-Dudek, 2018). Notwithstanding these concerns, conclusions were consistent with hypotheses and previous research. Noting concerns, future work could evaluate the current findings via a longitudinal study. The inclusion of multiple time points enables the observation of factors across time and ensures greater measurement consistency. This approach is beneficial to theory development because it will reveal the extent to which sleep-related states are temporally stable, and provide insights into the degree to which cognitive-perceptual personality factors, such as Auditory and Visual Hallucinations and preferential thinking style (subjective, intuitive, intra-psychic, etc.) interact with sleep-related states over time. Furthermore, use of longitudinal models enables the development of causal models.

A further potential limitation within the present study was the failure to screen for sleep-related conditions and psychiatric disorders. In the case of sleep-related conditions, researchers have linked narcolepsy with changes in dream mentation. Particularly, higher dream recall frequency and lucid dreaming (Dodet et al., 2015; Rak et al., 2015). Recent work has also reported an association between narcolepsy and creativity (Lacaux et al., 2019). Narcolepsy is a chronic sleep disorder characterized by excessive daytime sleepiness, disrupted nocturnal sleep, REM sleep occurring at the onset of sleep, and cataplexy (sudden loss of skeletal muscle tone in response to strong emotional stimuli) (Singh et al., 2013). Although narcolepsy is rare (1 in 2,000 people; Scammell, 2015) and therefore unlikely to have a significant effect on the results of this paper, subsequent research should screen for potentially conflating sleep-related conditions. In addition to this, future work could also control for psychiatric disorder. This is important because conditions such as psychosis can effect lucid dreaming (Mota et al., 2016; Voss et al., 2018) and predisposition to fantasy proneness and delusional beliefs (Tan et al., 2019). Moreover, these variables correlate positively with belief in the paranormal (Irwin et al., 2012a, b). In the current paper, these factors were unlikely to have influenced the reported outcomes because the sample was non-clinical. Regardless, it is important that future related work controls for these variables as they potentially influence incidence and experience of lucid dreaming.

Another possible limitation was the recruitment method used. The researchers advertised the study via emails to university staff/students and local stakeholders (businesses, leisure and vocational/sports classes), and invited only respondents who had experienced lucid dreams. In terms of sample composition, this approach has typically produced large data that were commensurate with equivalent studies (see Dagnall et al., 2014; Dagnall N. et al., 2016, Dagnall et al., 2019). Furthermore, there is no reason to believe that these samples are not reflective of the general population. This is especially true as the constructs indexed were psychological rather than ability based. Restricting selection to respondents who had experienced lucid dreaming was a prerequisite of the study aim, specifically the intention to examine how experience of lucid dreaming related to dissociated experiences related to REM sleep, proneness to reality testing deficits, and paranormal experiences/beliefs. Although this approach reduced variability with correlations, it avoided conflation by including participants who had not experienced lucid dreaming. In the case of the focal variable, lucid dreaming intensity, this is a major concern since there is a discrete difference between experiencing (absence vs. presence) and level (low to high). Combing these elements in analysis has a distorting effect on intensity by drastically reducing mean values. Noting these concerns, succeeding research should attempt to replicate outcomes with different more heterogeneous samples, and compare experiencers vs. non-experiencers of lucid dreams on study variables.

Overall, the present study provides a firm foundation for subsequent work on dissociated experiences related to REM sleep. This could consider incidence alongside factors such as control, intensity and content. Research might also usefully examine cultural and age-related differences.

## Data Availability Statement

The datasets generated for this study are available on request to the corresponding author.

## Ethics Statement

The studies involving human participants were reviewed and approved by The Manchester Metropolitan University Faculty of Health, Psychology and Social Care Ethics Committee. The patients/participants provided their written informed consent to participate in this study.

## Author Contributions

AD and ND focused theoretically, analyzed the data, and developed the article. KD collected the data and reviewed the draft.

## Conflict of Interest

The authors declare that the research was conducted in the absence of any commercial or financial relationships that could be construed as a potential conflict of interest.
